# The Increase in the Number of the Insane

**Published:** 1900-02-17

**Authors:** 


### The Institutional Workshop.

### THE INCREASE IN THE NUMBER OF THE INSANE.

Every year a vast pile of statistics on insanity makes its appearance, and when this mass is sifted it is seen with monotonous regularity that the preceding year has added several thousands to the already alarming bulk of our insane population. The first expression of opinion is invariably that this terrible disease is increasing among us; but it is soon pointed out that the general population of the country has also increased; that relieving officers hunt the lunatic more than they used to do; that the old dread of asylums is lessening among us; that the lives of the insane are longer now; and that the 4s. capitation grant has, like Eve, much to answer for, and so our fears are allayed as we carefully scan these explanations. With the first four of these postulates we need not say much, but with the fifth we feel inclined to quarrel. The number of workhouse lunatics is an ever-diminishing one, that of asylums an ever-increasing one. Most lunatics are more or less troublesome in workhouses, and as their cost of maintenance there, plus the establishment charges, is a mere trifle above the total asylum maintenance rate they are gradually being transferred. Deducting the capitation grant the asylum rate is positively lower than the workhouse rate, and this fact is likely to weigh with the guardians, who are naturally inclined to think more of the purely local rate than of the Imperial or county rate.

One enormous charge to the ratepayer is caused by the erection of asylums all over England; and it is certain that much unnecessary extravagance is permitted, if not actually encouraged, in these building operations. It has been proved that a very good asylum may be built for £150 per bed, and in spite of this fact we constantly hear of £200 and even more being spent. This is one of the things that ratepayers should look keenly into, and if Commissioners will not reduce their requirements, and if county councils will not resist extravagances, it behoves the public to take the matter into their own hands and memorialise the Home Secretary, and if necessary Parliament, to step in and prohibit the expenditure. There is one point that ratepayers should urge on all county councils, and that is the erection of departments in our county asylums for private patients. Accommodation should be provided for about 10 per cent. of the total provision for paupers. The profits would amount to a sum amply sufficient to keep the whole asylum in efficient repair, and so relieve the county rate, and the establishment charges would be distributed over a higher number, and so reduce the maintenance rate.

The important question now is, Can anything be done to reduce the number of pauper lunatics who are ever knocking at the gates of our county asylums in such increasing numbers? It is hard to say. There is so much human nature in men that their habits and passions and vices are not likely to yield to anything short of a universal crusade against them. If intermarriage of insane people were to be treated as a crime, and if drunkenness were to be bracketed with it in this respect, we should at once strike at the root of much of the insanity of our age; and, provided the punishment for breaking the law were severe enough, we should soon see an unprecedented reduction in the amount of insanity. But this can never be a party question; and we are not nearly civilised enough to hope for a coalition of parties to take it up in earnest. That legislation will one day be accomplished we hold to be certain, but we are not rash enough to predict when that day will be.

In the meantime the abolition of the capitation grant would do much to diminish the number of lunatics in asylums, and so would the introduction of the Scottish system of boarding-out. Of course, neither of these would decrease the actual number of the insane; but both would reduce the necessity for the ever-present building of asylums, and that is no small point. The obverse to this will occur to everyone conversant with asylums and workhouses, namely, that the patient is much more comfortable in the former than in the latter.

While there is no doubt that the numbers of the insane, as known to the authorities, are increasing rapidly, there is not much ground for believing that there is any great actual increase in the gross total of the insane. It is true that experts differ on this question, but it is admitted on all sides that the real increase of insanity is not, at present, an alarming one. We may pick up such grains of comfort as we can from this opinion, although we cannot help deploring the fact that insanity is not, like so many other diseases, regressing with the advance of learning. Practically it seems certain that more recoveries take place now among the acute cases than in the last generation, and we may feel certain that the next generation will suffer for our greater skill in this. We can hardly say that this is a comforting reflection, but we can boast of the number of cures in the present day.

